# Structural and Binding Effects of Chemical Modifications on Thrombin Binding Aptamer (TBA)

**DOI:** 10.3390/molecules26154620

**Published:** 2021-07-30

**Authors:** Vibhav Valsangkar, Sweta Vangaveti, Goh Woon Lee, Walid M. Fahssi, Waqas S. Awan, Yicheng Huang, Alan A. Chen, Jia Sheng

**Affiliations:** 1 Department of Chemistry, University at Albany, State University of New York, 1400 Washington Avenue, Albany, NY 12222, USA; vibhavvalsangkar@gmail.com (V.V.); lgw1452@gmail.com (G.W.L.); wawan@albany.edu (W.S.A.); huanyic1@gmail.com (Y.H.); 2 The RNA Institute, University at Albany, State University of New York, 1400 Washington Avenue, Albany, NY 12222, USA; svangaveti@albany.edu (S.V.); wfahssi@albany.edu (W.M.F.)

**Keywords:** thrombin binding aptamer (TBA), dendrimers, trebler, NHS-carboxy T

## Abstract

The thrombin binding aptamer (TBA) is a promising nucleic acid-based anticoagulant. We studied the effects of chemical modifications, such as dendrimer Trebler and NHS carboxy group, on TBA with respect to its structures and thrombin binding affinity. The two dendrimer modifications were incorporated into the TBA at the 5′ end and the NHS carboxy group was added into the thymine residues in the thrombin binding site of the TBA G-quadruplex (at T4, T13 and both T4/T13) using solid phase oligonucleotide synthesis. Circular dichroism (CD) spectroscopy confirmed that all of these modified TBA variants fold into a stable G-quadruplex. The binding affinity of TBA variants with thrombin was measured by surface plasmon resonance (SPR). The binding patterns and equilibrium dissociation constants (K_D_) of the modified TBAs are very similar to that of the native TBA. Molecular dynamics simulations studies indicate that the additional interactions or stability enhancement introduced by the modifications are minimized either by the disruption of TBA–thrombin interactions or destabilization elsewhere in the aptamer, providing a rational explanation for our experimental data. Overall, this study identifies potential positions on the TBA that can be modified without adversely affecting its structure and thrombin binding preference, which could be useful in the design and development of more functional TBA analogues.

## 1. Introduction

Hemostasis is an important process for all living organisms. When a blood vessel is damaged, clotting factors are released and they convert prothrombin into thrombin [1]. Thrombin is a serine protease that plays a major role in the coagulation cascade [2]. It converts the soluble protein fibrinogen into the insoluble fibrin, which further stabilizes platelet aggregates and leads to clot formation. However, when undesired coagulation occurs, it can result in serious conditions, such as deep vein thrombosis (DVT) and pulmonary embolism [3,4]. Anticoagulants are medications that help to prevent blood from over clotting. The current in-use anticoagulants, including warfarin, heparin and rivaroxaban [5,6], have several side effects, such as excessive bleeding, dizziness, hair loss, and tissue necrosis [7,8,9]. Thus, there is an urgent need for new anticoagulation therapies with enhanced efficacy and fewer side effects.

**Figure 1:**
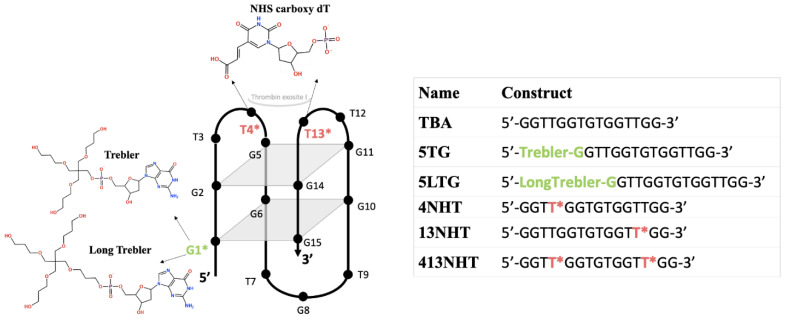
Modifications introduced in the thrombin binding aptamer and the different constructs used in this study.

An alternative to the existing anticoagulants, which are mostly small molecule drugs, is the use of aptamers. Aptamers are single stranded DNA or RNA that bind to their target molecules with high specificity and affinity [10]. Thrombin binding aptamer (TBA) is an example of a potential anticoagulant, that binds to thrombin specifically at the fibrinogen binding site in the protein (exosite I) and prevents the conversion of fibrinogen to fibrin and consequently clot formation. TBA is a 15-mer DNA oligonucleotide with the sequence of 5′-GGTTGGTGTGGTTGG-3′ and it self-assembles into an antiparallel G-quadruplex structure in solution with two G-quartets or G-tetrads—GT1 (G1, G6, G10, G15) and GT2 (G2, G5, G11, G14), as well as two T-T loops and one T-G-T loop (Figure 1). It is usually stabilized by a metal cation (Na^+^ or K^+^) in between the two quartets [11]. TBA binds to thrombin exosite Ⅰ via the T-T loops and exosite Ⅱ is a heparin binding site for the T-G-T loop [12,13,14,15]. Currently, one of the TBA variants is in clinical trials as an anticoagulant for patients undergoing coronary artery bypass graft surgery [16].

To improve the binding affinity between TBA and thrombin, several chemical and structural modifications have been explored such as TBA with 5-fluoro-2′-deoxyuridine, 5′-nitroindole, UNA (unlocked nucleic acid), LNA (locked nucleic acid), thiophosphoryl internucleotide linkages, or triazole internucleotide linkages [12,17,18,19,20,21,22]. Several of them maintain the native conformation and the ability of anticoagulation, but in certain cases the binding affinity dramatically decreased due to the structural disruptions that are introduced by the chemical modifications to the overall conformation of the TBA G-quadruplex. For example, when thiophosphoryl internucleotide bond was introduced in between the two G-tetrads, the thermostability of modified TBA decreased by up to 12 °C [21]. An exception was reported by Pasternak et al. such that a UNA-U in position T7 had higher thermodynamic stability, stronger binding affinity to thrombin, and better anticoagulant ability compared to the native TBA [19].

The binding affinity of native TBA to human α-thrombin has been reported to be 106 ± 5.1 nM [19]. In this study, we introduced two sets of modifications in the TBA, targeting to improve the binding between TBA and thrombin. As illustrated in Figure 1, we chose two dendrimer linkers on the 5′ end of TBA as our first set of modifications. These linkers are stable symmetric branched polymers with hydroxyl groups at their terminals named as Trebler (T) and Long Trebler (LT). Our initial hypothesis was that while the long arms of the linkers might be able to impart additional stability to the aptamer by wrapping around it, the terminal hydroxyl groups can provide additional hydrogen-bond interactions with the thrombin, thus improving the overall binding. For our second set of modifications, we chose the NHS (N-hydroxysuccinimide)-carboxyl group introduced at the C5 position of thymine. It was incorporated at the thrombin exosite I binding site of TBA in the TT loops, specifically at T4, T13 and both T4/T13 positions. NHS esters are one of the most popular amine-specific functional groups and bind with primary amine on protein to form amide bonds [23]. Exosite I of thrombin is lined with two Arg residues with primary amine groups that are involved in binding with TBA [13]. We hypothesized that NHS ester modifications in the binding site of TBA, namely the T-T loops, would interact with the amines and consequently have impacts on the binding between TBA and thrombin.

Our circular dichroism (CD) spectroscopy study confirmed that all of these modified TBA variants can fold into the same G-quadruplex structure as the native counterpart. Binding affinity studies by surface plasmon resonance (SPR) indicated that the interaction patterns and dissociation constants (K_D_) of the modified TBAs are very similar to that of the native one, although there was no discernable increase in binding. We further investigated the structural effects as well as the interactions of these modifications with thrombin through in silico modelling and molecular dynamics simulations studies, which indicate that the additional interactions or potentially stabilizing effects of the modifications are actually minimized either by the disruption of TBA–thrombin interactions or destabilization elsewhere in the aptamer, providing a rational explanation for our experimental observations. Overall, this study identifies potential positions on the TBA that can be modified without adversely affecting its structure and thrombin binding preference. In addition, our simulation studies also suggested new positions for future modifications that could be useful to the design and development of TBA analogues with improved functionality.

## 2. Materials and Methods

### 2.1. General Chemicals

Acrylamide, potassium chloride, sodium hydroxide, phosphate buffered saline (PBS), tetramethylethylenediamine (TEMED), and 10× TBE were purchased from Sigma-Aldrich. Then, 10 mg of 7.7 mg/mL human alpha Thrombin was purchased from Haematologic Technologies. Carboxyl sensor chip, activation buffer, blocking solution, 1-ethyl-3-(3-dimethylaminopropyl)-carbodiimide (EDC), and N-hydroxysuccinimide (NHS) for SPR were purchased from Nicoya Lifesciences.

### 2.2. Oligonucleotide Synthesis

Native TBA, Trebler modified TBA (T) and Long Trebler modified TBA (LT) sequences were chemically synthesized at 1.0-mol scales by solid phase synthesis using an Oligo-800 synthesizer. Commercially available native TBA was purchased from Integrated DNA Technologies (IDT). Dendrimer modifiers, Tris-2,2,2-[3-(4,4′-dimethoxytrityloxy)propyloxymethyl]-ethyl-[(2-cyanoethyl)-(N,N-diisopropyl)]-phosphoramidite and Tris-2,2,2-[3-(4,4′-dimethoxytrityloxy) propyloxymethyl]-methyleneoxypropyl-[(2-cyanoethyl)-(N,N-diisopropyl)]-phosphoramidite were purchased from Glen Research. Native phosphoramidites were purchased from ChemGenes Corporation and dissolved in acetonitrile with final concentration of 0.1 M. All the other reagents are standard solutions also obtained from ChemGenes. After synthesis, the oligos were cleaved from the solid support and fully deprotected with AMA (ammonium hydroxide:methylamine = 1:1) at 65 °C for 30 min. The amines were removed by Speed-Vac concentrator before purification. The DNA strands were purified by HPLC and characterized by denaturing polyacrylamide gel electrophoresis (PAGE).

### 2.3. Circular Dichroism Spectroscopy

CD spectra were measured by Jasco J-815 CD spectrometer by using a fused quartz cuvette with 1 mm of light path length. The data was collected at 25 °C in the range of 220–320 nm with 1.00 nm bandwidth and data pitch. Spectra were collected with 3 accumulations and at a 100 nm/min scanning speed. No baseline correction was done, but the spectra were smoothened with convolution width of 25 by Savitzky–Golay method. Then, 5 µM solutions of native TBA and modified TBA were prepared in 10 mM KCl and annealed at 95 °C for 5 min and cooled down to room temperature to allow the structure to refold. Further, 100 µM solution of Thrombin was also prepared in 10 mM KCl. All plots were generated with SigmaPlot 12.0.

### 2.4. Surface Plasmon Resonance (SPR) Binding Study

A carboxyl sensor chip was used for the experiment. Briefly, 50 µg/mL of Thrombin was prepared in the activation buffer. Different concentrations of modified TBA (except 5′ FAM modifications), with 1, 2, 4, 8, and 16 µM analyte samples, were prepared in the running buffer, 0.005% PBS TWEEN 20 10 mM KCl pH 7.4, then annealed at 95 °C for 5 min and cooled down to room temperature to allow the structure to refold. Binding of modified TBA to Thrombin was studied with OpenSPR, a benchtop version of SPR from Nicoya Lifesciences. First, the carboxyl sensor chip was washed with 80% isopropanol (IPA) and dried with air. Priming the SPR system, taking new reference spectra, and filling the flow cell were undertaken prior to experiment. To remove any air bubbles in the flow cell and tubes, 80% IPA was injected at the maximum flow rate 150 µL/min. To activate the carboxyl sensor chip, to clean the surface, and to block nonspecific binding, EDC/NHS mixture (thaw and mix immediately), 10 mM HCl, and blocking agent were injected to open SPR respectively at the lowest flow rate of 20 µL/min. For regeneration, 5 mM NaOH was injected at flow rate 150 µL/min. After changing the flow rate to 20 µL/min, 1 µM modified TBA was injected and left to dissociate for 6 min or more. Before every injection of the next analyte, 5 mM NaOH was injected to remove any unbound TBA at the maximum flow rate for 150 µL/min. The TBA analyte samples were injected in increasing order. SPR data were kinetically analyzed by TraceDrawer software.

### 2.5. Molecular Modelling and Simulation Methods

To understand in detail the structural effects of the modifications on TBA, molecular dynamics simulations were performed. First, AMBER [24] type force-field parameters were developed for the modifications. The geometries of the modified nucleosides were optimized using Hartree–Fock level theory and 6-31G* basis-sets. Partial charges on the atoms were then obtained using the online RESP charge-fitting server R.E.D.D [25,26]. AMBER-99 force-field parameters with bsc1 modification [27] were used to generate bonded and non-bonded interaction parameters for the modified nucleosides. Rotamer libraries for the modifications were generated in MOE [28]. The initial structure of thrombin bound to the unmodified TBA (PDB ID: 4DII) was downloaded from the protein data bank [29]. For our MD studies, five structures of the modified TBA bound to thrombin were generated using the initial unmodified structure and the rotamer library generated in MOE—three with the NHS-Carboxy T modification at the 4th (4NHT), 13th (13NHT), and the 4th and 13th (413NHT) position of the aptamer, and two with the short (5TG) and the long trebler group (5LTG) at the 5′ end of the TBA. A total of 12 initial structures were simulated, namely the native, 4NHT, 13NHT, 413NHT, 5TG, and 5LTG, for the thrombin free and thrombin bound aptamer.

Molecular dynamics simulations were performed using GROMACS 2019.4 [30] on all twelve systems in a solution of 0.01 M KCl in a cubic box. The size of the box and the number of ions and water molecules for the aptamer simulations were: 6.14 nm containing 15 K^+^ and 1 Cl^−^ ions and ~7503 water molecules. For the thrombin bound simulations, a box of size ~9 nm containing 14 K^+^ and 1 Cl^−^ ions and 6136 water molecules was used. The MD simulations incorporated a leap-frog algorithm with a 2-fs time step to integrate the equations of motion. The system was maintained at 300 K, using the velocity rescaling thermostat [31]. The pressure was maintained at 1 atm using the Berendsen barostat for equilibration [32,33]. Long-range electrostatic interactions were calculated using the particle mesh Ewald (PME) algorithm with a real space cut-off of 1.0 nm [34]. Lennard–Jones interactions were truncated at 1.0 nm. The TIP3P model was used to represent the water molecules, and the LINCS algorithm was used to constrain the motion of hydrogen atoms bonded to heavy atoms [35]. The system was subjected to energy minimization to prevent any overlap of atoms, followed by a short equilibration (0.3 ns) and 100-ns production run. Coordinates of the DNA and protein were stored every 2 ps for further analysis. The simulations were visualized using Visual Molecular Dynamics (VMD) software [36] and analyzed using tools from GROMACS [30]. Hydrogen bonding analysis was performed in VMD using a donor-acceptor distance cutoff 0.33 nm and the hydrogen–donor–acceptor angle cutoff of 30 degrees.

To propose new modifications that can potentially enhance thrombin–aptamer interactions, the minimized structures from the simulation were analyzed in MOE. The interaction site was explored for the possibility of expanding the modification or adding functional groups to introduce new hydrophobic and/or hydrophilic interactions between thrombin and the aptamer. The interaction strength was then calculated using a local minimization of the modification based on AMBER 10:EHT included within MOE [28].

## 3. Results

### 3.1. Modified TBA Constructs Maintain Native TBA Folding

**Figure 2:**
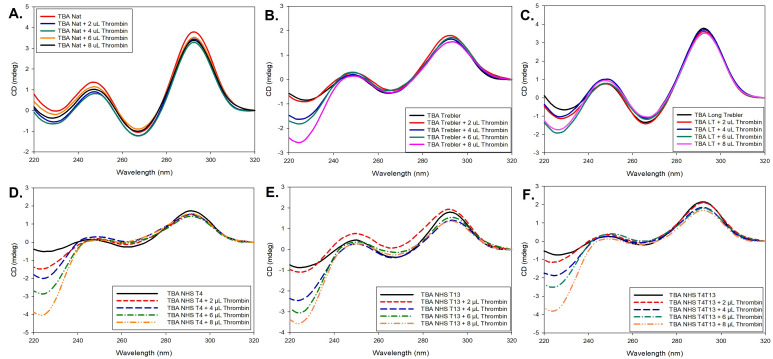
CD spectra of (**A**) TBA, (**B**) 5TG, (**C**) 5LTG, (**D**) 4NHT, (**E**) 13NHT and (**F**) 413NHT titrated with increased equivalence of 100 µM thrombin solution.

The native TBA folds into an antiparallel G-quadruplex structure that can be identified by its signature CD spectra profile in the wavelength range of 220 nm to 320 nm, with a moderate positive peak at ~245 nm, a negative peak at ~267 nm, and an intense high peak at ~293 nm [37], as shown in Figure 2A. To check for any changes in confirmation of the aptamer upon protein binding, thrombin was added in increments of 2 µL from 100 µM Thrombin stock solution (200 pmoles at each increment) to the aptamer (1 nM), and the corresponding CD spectra were collected. The experimental conditions, such as concentration of the aptamer, Thrombin, and 10 mM KCl buffer, were finalized based on initial trials at different concentrations as well as buffer conditions. The same process was repeated for the different modified constructs. As shown in Figure 2, for each case, as the amount of added thrombin increases, the intensity of the peaks is slightly affected, but there is no shift in the peak wavelengths, indicating that the overall G-quadruplex structure is well maintained across all modifications both in the absence of thrombin and upon thrombin binding, despite some small local perturbations. More specifically, the band intensities for the 5TG construct are relatively lower compared to the native TBA as shown in Figure 2B. Though the spectra show patterns similar to that of the G-quadruplex, there were intensity shifts in the range of 220–240 nm as a sign of local structural switches. With the increasing amounts of thrombin, the 5TG can maintain its stable antiparallel G-quadruplex structure in the process. In the case of the longer trebler group, 5LTG (Figure 2C) and NHS constructs (Figure 2D–F), the overall band intensities are close to the native TBA and similar intensity shift is observed as the 5TG case at lower wavelengths.

### 3.2. Binding Affinities of Modified TBA Constructs Are Comparable to Native TBA

**Figure 3:**
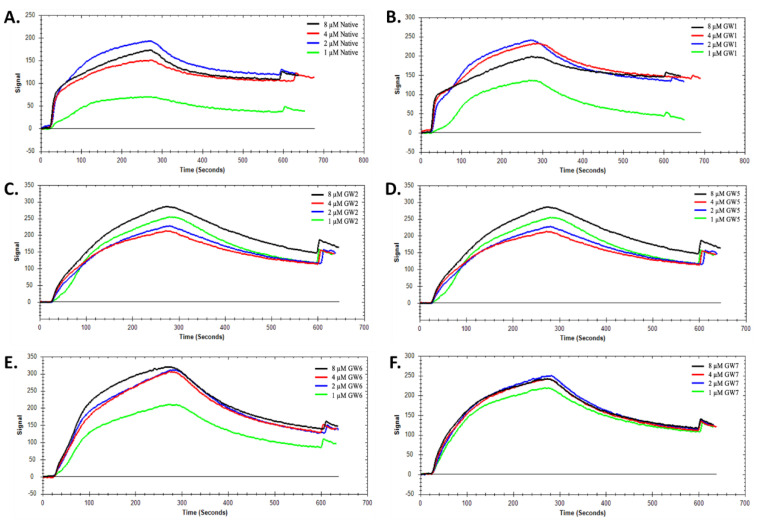
SPR kinetic analysis of (**A**) native TBA, (**B**) 5TG, (**C**) 5LTG, (**D**) 4NHT, (**E**) 13NHT, and (**F**) 413NHT TBA constructs.

To measure the binding constants using SPR, the thrombin molecule was immobilized on the carboxyl-coated sensor chip by the interactions with amine groups from Arg, Tyr, Lys and Ile residues. Before the measurement, the blank binding test of TBA with the sensor chip was performed to eliminate the non-specific binding between TBA and chips. After immobilization of thrombin, different concentrations of TBA solution ranging from 1–8 µM were added to the complex for SPR analysis. The original binding curves are shown in Figure 3 and the binding constants (K_D_) are summarized in Table 1.

The kinetic analysis was conducted using 1:1 binding model on the TraceDrawer software provided by Nicoya Lifesciences. The average dissociation constant (K_D_) value of native TBA was calculated as 99.8 nM, which is consistent with the previous literature value, 102.6 ± 5.1 nM [19]. Under the same condition, the average dissociation constant (K_D_) values of 5TG and 5LTG were 102 nM and 99.6 nM respectively. These close K_D_ values for the TBAs with the 5′ trebler modifications indicate that these modifications do not alter the overall interactions between TBA and thrombin. Similarly, the 4NHT, 13NHT, and 413NHT TBA constructs were also applied under the same conditions. Saturation of binding was observed at the concentration of 8 µM in Figure 3d–f and the K_D_ values for 4NHT, 13NHT and 413NHT TBA were 99 nM, 97.5 nM, and 99.8 nM, respectively, with similar signal trend as the native TBA. A previous study that used unlocked nucleic acids (UNA) at various positions of the TBA [19] showed that a UNA at the 4th position decreases binding affinity of TBA to thrombin (Table 1), which was attributed to the flexibility of the UNA that leads to the disruption of either the G-tetrad itself or TBA’s interaction with thrombin. In the case of 4NHT, however, we observe that, for a modification at the same position, the change in K_D_ is insignificant. These observations together indicate that the modifications investigated in this study maintain native TBA-like binding pattern and affinity to thrombin.

### 3.3. Molecular Dynamics Simulations of TBA and Its Modified Constructs Agree with CD Data

**Figure 4:**
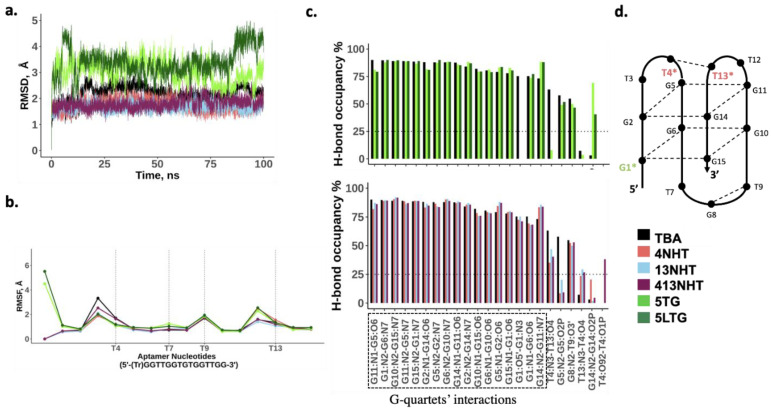
MD simulation results for the thrombin free aptamers. (**a**) Root mean square deviation from the initial structure; (**b**) Average root mean square fluctuations for each nucleotide in the native and modified aptamer. Trebler group is treated as a separate nucleotide; (**c**) Bond occupancies for intra-hydrogen bonds in the aptamer. Only H-bonds with at least 25% occupancies are shown (**d**) Schematic showing h-bond interactions within the aptamer.

In order to understand the biochemical observations of the effect of the modifications on TBA, we performed molecular dynamics simulations of the TBA with and without the modifications. Our simulations of the thrombin free aptamer agree with the observations of the CD experiments described above. All the constructs of the TBA maintain a stable G-quadruplex structure in the absence of thrombin. As shown in Figure 4a, all-atom root mean square deviation (RMSD) of all the structures from the initial folded structure is small (<5 Å). The root mean square fluctuations (RMSF) of the nucleotides involved in the G-quartets are small, and the higher values coincide with the TT and TGT loop nucleotides, and the trebler modified 5′ G (Figure 4b). Most native TBA interactions within the aptamer are also maintained in the modified constructs as is evident from the comparable hydrogen bond occupancies within the G-quartets for the modified and native TBAs (Figure 4c). However, a few differences are observed. In the case of the trebler modifications, the flexible trebler group on the 5′ end (5TG, 5LTG) prevents the H-bond interaction within the G1 nucleotide between its O5′ and N3 atoms, that is present in the unmodified TBA but an additional interaction is observed within G14 between the nucleobase and the backbone. While these interactions can potentially offset each other’s effects, it is interesting to note that the modification in the 5′ end adversely affects the interaction between T4 and T13 on the other end of the aptamer, suggesting allosteric effects are in play. The NHT modifications promote one additional h-bond interaction between T4 and T13, which enhances its stacking to the adjacent quartet (GT2), leading to a slightly more stable quartet as seen by the small increase in H-bond occupancies for G11–G14 and G2–G5 pairs.

### 3.4. Molecular Dynamics Simulation of Thrombin Bound TBA and Its Modified Constructs Agree with SPR Experiments

**Figure 5:**
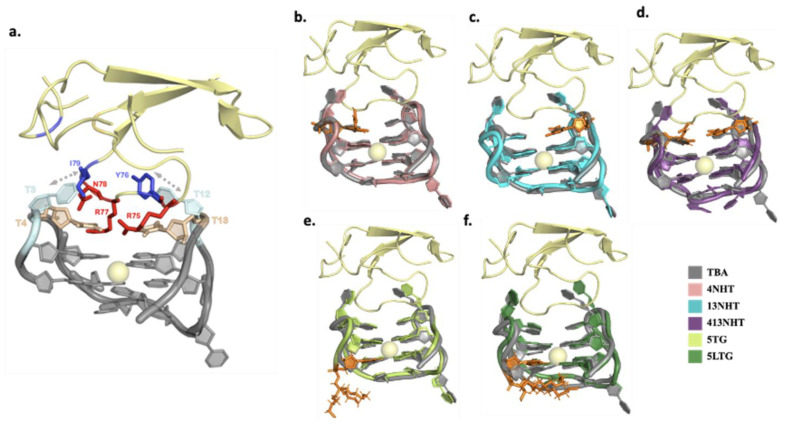
Structures of thrombin bound aptamers. The protein and the potassium ion are shown in yellow and the unmodified aptamer in gray (**a**) The aminoacid residues and nucleotides involved in the interaction between thrombin and the unmodified aptamer (hydrophobic interactions in shades of blue, and hydrophilic in red and wheat) (**b**–**f**) Structures of the dominant conformation from the simulations for the modified aptamer systems overlaid on the unmodified aptamer (gray). The modified nucleotides in each case are highlighted in orange.

**Figure 6:**
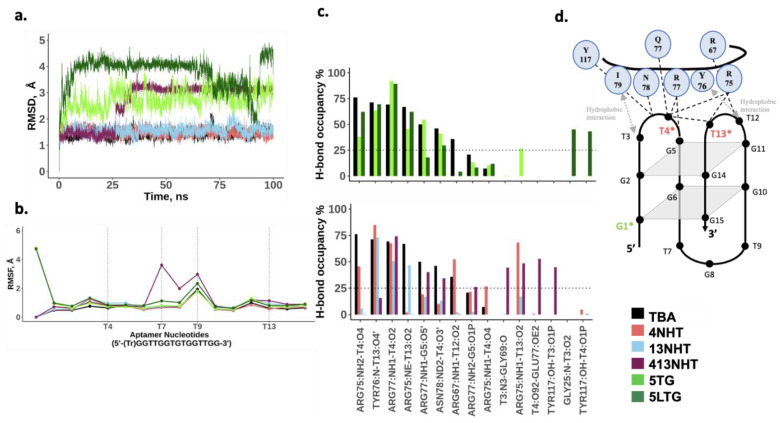
MD simulation results for the thrombin bound aptamers. (**a**) Root mean square deviation from the initial structure; (**b**) Average root mean square fluctuations for each nucleotide in the native and modified aptamer. Trebler group is treated as a separate nucleotide; (**c**) Bond occupancies for inter-hydrogen bonds of the aptamer with thrombin. Only H-bonds with at least 25% occupancies are shown; (**d**) Schematic of the various interactions between the aptamer and thrombin.

Another set of simulations of thrombin bound TBA were performed to understand the effects of modifications on the interaction of TBA with thrombin in structural detail. The RMSD (Figure 5 and Figure 6a) for the aptamer bound to thrombin is similar to that of the thrombin free aptamer, suggesting that the structure does not deviate from its initial configuration upon thrombin binding. In these simulations, the RMSF of TT loops which are on the thrombin binding side of the G-quartets in the aptamer, is lower than in the thrombin free aptamer simulations. This is expected as the aptamer interacts with thrombin via these loops thus stabilizing the nucleotides. Surprisingly, for the 413NHT construct, we observe that the TGT loop on the other side of the G-quartets has a higher RMSF compared to all other variants (Figure 6b). To check the reproducibility of this unexpected effect, we ran twenty simulations of the system with the 413NHT construct. We observe that indeed the TGT loop has a high RMSF and thus appears to be destabilized by the NHT modifications in the TT loops (Supplementary Figure S1). This alludes to the interdependence between the TT and TGT loops of the aptamer where modifying one affects the stability of the other.

Next, we analyzed the interface of the thrombin–aptamer complex. The interaction between TBA and thrombin consists of both polar (hydrogen bonds) and non-polar interactions and has been reported in detail in several previous studies [38]. Specifically, the TT loops of TBA interact with thrombin via a loop region formed by amino acid residues 74–80, with T3 and T12 forming a pincer-like structure, while T4 and T13 establish hydrogen bonding with the loop amino-acid side chains that occupy the space between the TT loops in close contact with the aptamer. The interactions include: (i) hydrophobic contacts between T3 and Ile74, (ii) stacking interactions between T12 and Tyr76, (iii) hydrogen bonds between T4, T13 and Arg75, Arg77 of thrombin (Figure 5a). In our simulations, we observe that the modifications did not significantly disrupt the previously reported interactions between thrombin and TBA, which is in agreement with the experiments—the dissociation constants listed in Table 1, for the different modified variants of TBA, are comparable to the unmodified TBA. The hydrophobic interactions are maintained throughout the simulation in all the modified variants of TBA. The most robust hydrogen bond interaction is that between one of the terminal amines of Arg 77 and the carbonyl oxygen O2 on T4 which is not affected by the presence of modifications. Arg77 is also held in position with a supporting interaction between the other terminal amine on Arg 77 and the backbone of G5. The interactions of the aptamer with residue Arg 75 are more prone to be affected by the modifications. Arg75 is positioned such that it interacts with both T4 and T13 in the native TBA. In the presence of the NHT modifications, Arg 75 adapts a configuration different from the native, such that the native contacts are either aided by additional interactions or replaced by a different set of similar hydrogen bond interactions (Figure 6c), thus leading to a marginal improvement in the thrombin binding capacity of TBA. Interestingly, in case of the long trebler modification, additional interactions are observed between the T3-T4 loop nucleotides and amino acid residues Gly25 and Tyr117. The long trebler group, however, does not have any direct contact to facilitate this interaction, suggesting allosteric effects that were also observed in the case of the free aptamer simulations. However, the effect of any new hydrogen bonds observed in the modified constructs is offset by the disruption of hydrogen bond interactions of the native TBA with thrombin, thus having no significant influence on the binding of the TBA to thrombin, as noted in Table 1.

## 4. Discussions

In this work, we introduced modifications on three locations in the TBA and investigated the effect of the modifications on aptamer conformation and thrombin binding ability. In our CD experiments, we observed that the modifications do not alter the G-quadruplex fold of TBA, which was promising, since the quadruplex fold is important for its interaction with thrombin, as previously reported [11]. Several previous studies have focused on optimizing the structural stability of the G-quadruplex of the TBA by the use of Fluorine modified bases [17], locked/unlocked nucleic acids [19,39,40], etc. These approaches have been able to enhance the stability of the G-quadruplex in specific cases but have not been as successful in improving the binding affinity of TBA with thrombin. In our approach, we hypothesized that the introduction of the dendrimeric and two NHS carboxy modifications at G1, T4, and T13 of the aptamer, respectively, would boost the binding affinity of TBA to thrombin by introducing additional hydrogen bonding interactions.

Our subsequent SPR experiments to measure the binding affinity of aptamer to thrombin, however, showed that thrombin binding is not affected by these modifications. This was surprising and contrary to our working hypothesis. We performed molecular dynamics simulations to investigate this further. Overall, the MD simulations were in agreement with the experiments. In the simulations of the free aptamer, we observed that none of the modified constructs deviated from antiparallel G-quadruplex structure of the unmodified aptamer. In case of the thrombin bound aptamer simulations, however, we note subtle differences in the overall stability of the aptamer and its interaction with thrombin in the presence of the modifications. The NHS-carboxy T modifications on the TT loops at the thrombin binding interface affect the fluctuations of the TGT loop on the opposite end of the aptamer. In our constructs, the destabilizing effect was not substantial enough to adversely affect thrombin binding. However, this suggests that when introducing modifications in the aptamer, their effects on the loops on either end of the G-quartets must be taken into account to ensure the intended outcome. In terms of the actual interaction with thrombin, all the modifications that we studied were well tolerated in the aptamer in their respective positions. Any disruption in existing thrombin–aptamer interactions in the presence of the modifications was compensated by additional interactions formed elsewhere within the structure, thus illustrating the robustness of TBA–thrombin interaction.

Since we observed in our simulations that the interactions between one of the interfacial arginines (Arg 75) and TBA are dynamic and affected the most by changes to the TBA, we used in silico modelling to explore additional modifications at T4 and T13 of the aptamer that could improve local interactions near the binding site. Our preliminary findings suggest that introducing small changes to the NHS carboxy group in the TT loops can potentially enhance thrombin–aptamer interactions. For example, a carbonyl group added to the second carbon of the NHS carboxy group on T4 not only allows for an additional interaction with Arg75, but also increases its flexibility, allowing the carboxyl group to interact with the phosphate backbone and provide ancillary support to hold T3 in a favorable position (Supplementary Figure S2). The modelling results are encouraging and testing the proposed modifications in vitro is part of our ongoing and future work.

## 5. Conclusions

The thrombin binding aptamer has shown a higher efficiency in clinical trials compared to the existing small molecule anticoagulants like heparin. However, it requires a very high dosage to attain the desired levels of anticoagulation. A number of studies have since focused on improving the binding efficiency of the aptamer to thrombin as a potential strategy to lower the dosage and improve efficacy. Neither the NHS carboxy T modifications at the thrombin binding interface nor the dendrimer linkers on the other 5′ end of the aptamer improve thrombin binding, but at the same time they did not destabilize the structure of the aptamer either. Using in silico modelling and simulations, we have gathered insights concerning reasons for the ineffectiveness of our modified constructs and we also have ideas for the type of modifications that can have the intended effect of increased thrombin binding ability of TBA. Moving forward, we will use a combined approach of computational modelling and experiments to design effective modified aptamers.

## Figures and Tables

**Table 1:** Dissociation constant values (K_D_) for the different constructs.

Name of the Construct	Dissociation Constant K_D_ (Average Values Calculated from Our SPR Experiments) (nM)	Reported K_D_ (from Literature [19] for Unmodified/Modified TBA at Same Locations) (nM)
TBA	99.8 (±8.96)	102.6 ± 5.1
5TG	102 (±2.22)	-
5LTG	99.6 (±1.2)	-
4NHT	102 (±2.21)	172.3 ± 8.1 ^a^
13NHT	99.7 (±1.34)	-
413NHT	102 (±2.22)	-

^a^ The values reported in this study are for an unlocked nucleic acid (UNA) modification at the 4th position.

## Data Availability

Not applicable.

## Supplementary Materials

The following are available online, Figure S1: Root mean square fluctuations (RMSF) for the 413NHT construct compared with the unmodified construct (TBA). Figure S2: Licorice representation of original NHT Carboxy and modified NHO Carboxy along with their interaction diagrams.
