# Supplementary material for: Structural and Binding Effects of Chemical Modifications on Thrombin Binding Aptamer (TBA)
Source: Molecules. 2021 Jul 30;26(15):4620. doi: 10.3390/molecules26154620 (PMC8348300; doi:10.3390/molecules26154620)
Supplement: Supplementary file 1 [file molecules-26-04620-s001.zip › molecules-1297085-supplementary.pdf]

Supplemental Information : Structural and Binding Effects of Chemical Modifications on Thrombin Binding Aptamer (TBA)

Vibhav Valsangkar, Sweta Vangaveti, Goh Woon Lee, Walid M. Fahssi, Waqas S. Awan, Yicheng Huang, Alan A. Chen, Jia Sheng

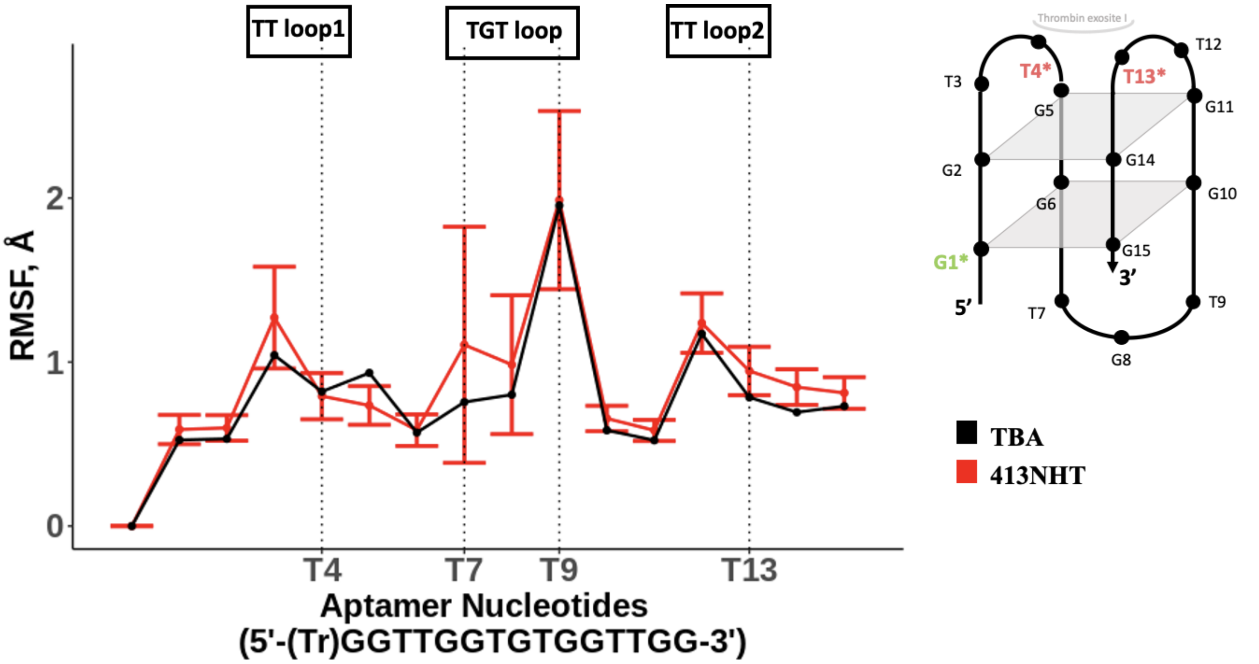

Figure S1. Root mean square fluctuations (RMSF) for the 413NHT construct compared with the unmodified construct (TBA). For the 413NHT construct (red), the average RMSF and the standard deviation (represented as error bars) were calculated from 20 replicate simulations of the thrombin bound 413NHT construct at 315K. The replicates all use the same starting structure but different initial velocities.

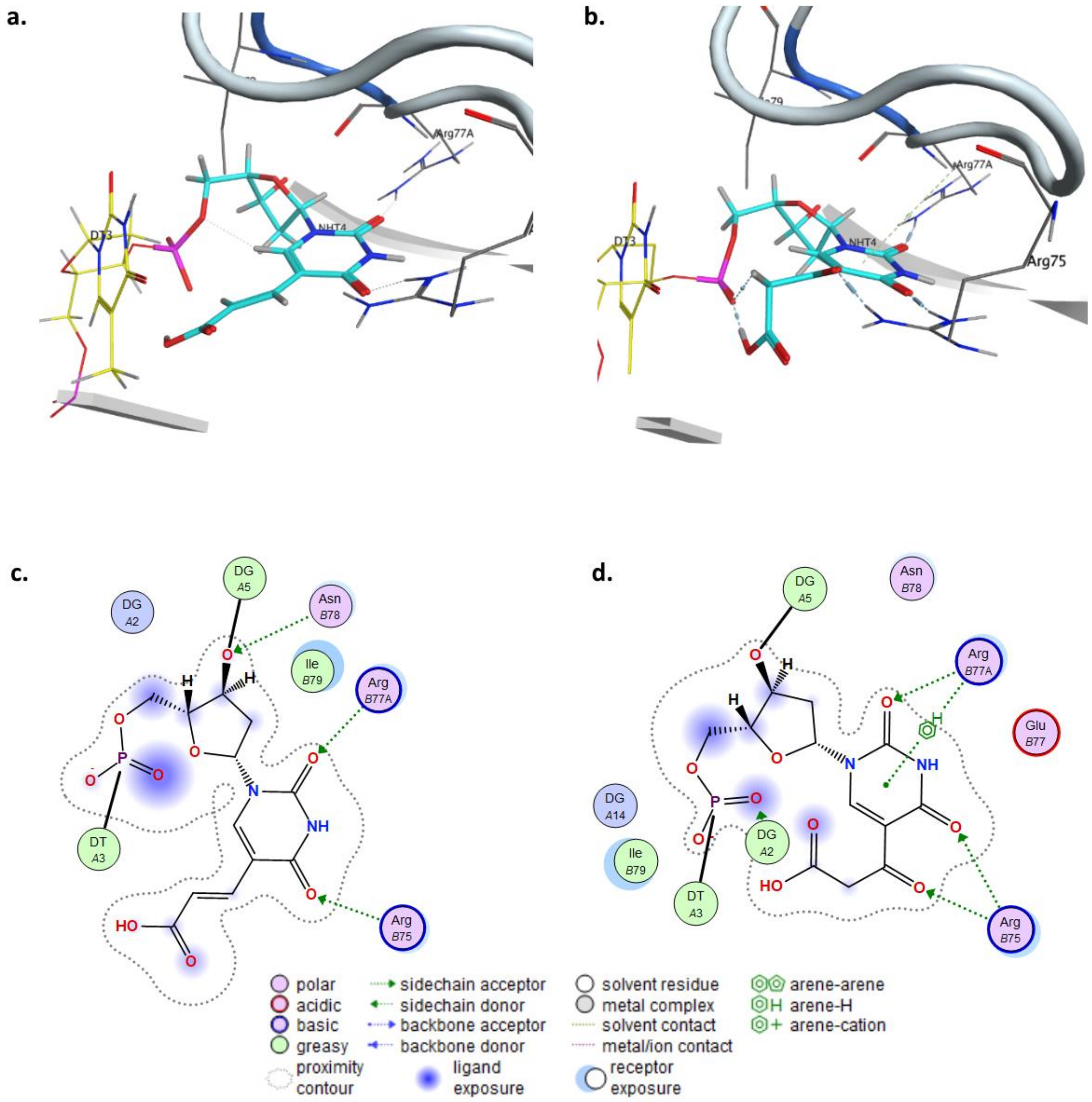

Figure S2. Licorice representation of original NHT Carboxy **(a)** and modified NHO Carboxy **(b)** along with their interaction diagrams in **(c)** and **(d)** respectively.

(a, c) The carboxyl group on NHT is observed to jut out and push against the hydrophobic ring of T3. Arg75 interacts with the para carbonyl on NHT4 via one hydrogen bond. **(b, d)** The carboxyl group in NHO folds downwards (due to added stability achieved by removing the double bond) and interacts with the non-bridging oxygens in the phosphate backbone and allows the hydrophobic areas of T3 and the carbon chain to interact favorably. This conformation also allows an additional hydrogen bond between the newly introduced carbonyl to interact with Arg75.
